# Eight Years of Severe Allergic Reactions in Finland: A Register-Based Report

**DOI:** 10.1097/WOX.0b013e3181898224

**Published:** 2008-11-15

**Authors:** Soili Mäkinen-Kiljunen, Tari Haahtela

**Affiliations:** 1 Skin and Allergy Hospital, Helsinki University Central Hospital, PO Box 160, 00029 HUS, Helsinki, Finland

**Keywords:** allergen preparation, anaphylaxis, drug, food, insect, register

## Abstract

**Background**

No data have been available on severe allergic reactions in Finland.

**Materials and Methods**

We summarize the data accumulated from 2000 to 2007 in the national register established at the Skin and Allergy Hospital of the Helsinki University Central Hospital, where physicians voluntarily report on patients with severe allergic reactions.

**Results**

During the period, the 530 reported cases of severe allergic reactions represented an annual frequency of 0.001%. Of the patients, 66% were adults and 56% were female, with a median age of 27 years. Food was the causative agent in 53% of the cases, drugs in 26%, allergen preparations in 12%, and insects in 8%. Dermatologic symptoms were reported in 85%, respiratory in 76%, cardiovascular in 50%, gastrointestinal in 33%, and eye/nose symptoms in 18%. The reaction was a life-threatening anaphylactic shock in 26% of the cases, with no deaths reported. Patients were treated with intramuscular adrenaline in 75% of the cases. Not only nuts and seeds, but also fruit and vegetables were the most important allergens for the adults. Nuts were also important allergens for children, along with milk, egg, and wheat. In addition, many "exotic" allergens were identified: patent blue, carmine dye, yeast, buckwheat, and macrogol.

**Conclusions**

Severe allergic reactions are underreported, but a register reflects the real-life situation and helps to identify new causative agents. It also contributes to improvements in first aid treatment practice.

## 

Anaphylaxis is an immunoglobulin E (IgE)-or non- IgE-mediated, severe, life-threatening systemic allergic reaction, with a rapid onset and multiple organ system involvement [1,2]. Independent of the mechanism, the first aid treatment of choice is intramuscular adrenaline. Because of the risk of recurrent severe reaction, the causative allergen must be recognized and avoided when possible.

To our knowledge, nationwide anaphylaxis registers or similar organizations exist at least in Denmark, France-Belgium, Germany-Austria-Switzerland, Italy, the Netherlands, Norway, Sweden, the United Kingdom and Ireland, and also in the United States (Table 1) [3-7]. Maintaining a comprehensive anaphylaxis register involves several difficulties, for example, the lack of internationally accepted clinical definition of anaphylaxis has made it difficult to diagnose an anaphylactic reaction (Table 2).

**Table 1:** Anaphylaxis Registers and Anaphylaxis Organizations

Country	Register Type and Contact Address	Responsible Informant/Organization
Several	The Food Allergy and Anaphylaxis Alliance	Since 1999, allergy network groups from the United States, United Kingdom, Canada, Quebec, New Zealand, Italy, Australia, and the Netherlands.
	A voluntary registry for peanut and tree nut allergy (United States, since 1997)	
Denmark	Danish Anaesthesia Allergy Centre	Clinicians may refer their patients to Danish Anaesthesia Allergy Centre for further investigation
Finland	National anaphylaxis register	1-Page anaphylaxis form to be filled by physician
France and Belgium	French Allergy Vigilance Network (Available at: www.cicbaa.org)	1-Page declaration of severe allergic accident to be filled in mainly by French and Belgian allergists
	The Study Group on Anaphylaxis during Anaesthesia (GERAP)	
Germany, Austria, and Switzerland	Deutscher Allergie-und Asthmabund (Available at: www.anaphylaxie.net)	Patient organization reports from clinicians
Norway	The Norwegian National Reporting System and Register of Severe Allergic Reactions to Food (Food Allergy Register)	Government-funded system, 1-page reporting form from physicians, IgE and allergen analysis service
Sweden	Swedish system for reporting severe and fatal reactions caused by food	National system for reporting by the medical profession
United Kingdom	The United Kingdom anaphylaxis register	Since 1992 for fatal anaphylactic reactions in the United Kingdom

FAAA indicates Food Allergy and Anaphylaxis Alliance.

**Table 2:** Difficulties in Maintaining an Anaphylaxis Register

Problem	Solution	Suggestion
1. Lack of definition of anaphylaxis: anaphylactic reaction vs anaphylactic shock	Clinical diagnosis [17]	Combination of circulatory, dermatologic, respiratory, gastrointestinal symptoms
2. Not all reactions are reported	Improvement of knowledge and vigilance system	FAAA Ga2len network
3. Not all allergens are considered	Exchange of register information	FAAA Ga2len network
4. Allergen not identified	More resources, better organized testing	All anaphylaxis patients should be seen by an allergologist

FAAA indicates Food Allergy and Anaphylaxis Alliance.

No data reveal actual frequency of severe allergic reactions in Finland. The literature has reported incidence figures of 7.9 to 21 anaphylaxis cases per 100,000 person-years,[8-10] along with increasing allergy prevalence and hospital admission figures during the past decade [11-13]. In the United States, approximately 30,000 cases of food anaphylaxis occur annually, 150 to 200 of which are fatal [14]. In the United Kingdom, the incidence of fatal reactions is one in 3 million person-years, and its National Anaphylaxis Campaign was set up in 1994 for guidance and information on allergens and allergic reactions [15-17].

In 2000, the Finnish register of severe allergic reactions was established at the Skin and Allergy Hospital of the Helsinki University Central Hospital, where physicians--mostly allergologists--voluntarily report patients with severe allergic reactions independently of the causative agent. A 1-page questionnaire form is available on the Internet.

## Materials and methods

We summarize the first 530 cases reported to the register between 2000 and 2007. Our results are based on the anonymous patient data provided by the physicians in the questionnaire form evaluated by an allergy specialist.

Any adverse reactions from vaccines that were reported to the National Public Health Institute are absent from the present article.

## Results

### Reports on severe allergic reactions

A total of 530 cases of severe allergic reactions were reported between 2000 and 2007 throughout the country, mostly (65%) from the Helsinki capital area, which represents 25% of the entire population of 5.2 million. This represents an annual incidence of 0.001%.

### Patients

Of the 530 cases, 348 (66%) were adults and 182 (34%) were children younger than 16 years. Females accounted for 295 (56%). Median age was 27 years; range was from 2 months to 92 years. Two peaks in age distribution were for children younger than 5 years and young adults aged 25 to 35 years.

A previously diagnosed allergy occurred in 91%; skin disorders were present in 43%, allergic rhinoconjunctivitis in 43%, asthma in 33%, and gastrointestinal disorders in 6%. A previous anaphylaxis affected 23%.

The reactions were regarded as severe anaphylactic shock reactions in 26% of the cases, with a systolic blood pressure in adults less than 90 mm Hg or with severe respiratory symptoms such as bronchospasm and upper airway angioedema [18]. Other severe allergic reactions included nonshock reactions with symptoms in at least two of the following organs: skin, respiratory tract, cardiovascular system, or gastrointestinal tract [18].

### Symptoms and treatment

In 74% of the cases, the first symptoms predicting severe allergic reaction appeared less than 30 minutes after contact with the inciting allergen.

Skin symptoms were most commonly reported, in 85%, followed by respiratory (76%), cardiovascular (50%), and gastrointestinal (33%) symptoms.

Most frequently, corticosteroids were used for treatment, in 84%, followed by adrenaline in 75%, and antihistamine in 67%. Of the adrenaline-treated patients, 10% used a ready-to-use adrenaline autoinjector.

### Allergens and exposure

**Figure 1:**
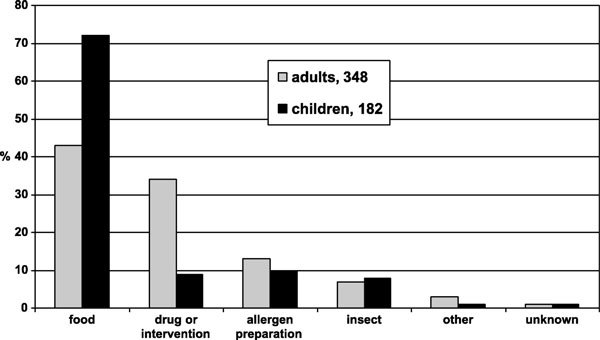
**Causes for severe allergic reactions (%) in adults and in children between 2000 and 2007 in Finland**.

Foods were the causative agent in 279 (53%) cases, drugs and medical interventions in 136 (26%), allergen preparations for diagnostic work and immunotherapy in 64 (12%), and insects in 40 (8%) (Figure 1). In 13 patients (2%), other causes were noted, and only 6 cases (1%) were idiopathic or without any recognizable cause (Table 3). In 7 cases, both food and a drug were suspect causative agents. An additional known or suspected factor in 29 cases (5%) was exercise.

**Table 3:** Allergens Causing Severe Allergic Reactions in Finland

Allergen	Adults	Children	Examples of Specific Agents
Foods	148	131	
Nuts/seeds	38	21	
Tree nuts	21	10	Hazel (n = 5), cashew (n = 3), walnut (n = 3), almond (n = 3; marzipan, n = 1), pistachio (n = 2)
Seeds	12	3	Sesame (n = 5; tahini, n = 3), pine (n = 3), flaxseed (n = 2), mustard
Peanut	5	8	Cooked peanut taken as beans
Fruit	19	12	Kiwi (n = 8), apple (n = 5), banana (n = 5), grape (n = 3), mango (n = 4), lychee, jackfruit, plum (n = 2), red currant
Vegetables	11	5	Celery (n = 5), carrot (n = 4), parsnip (n = 2), pea (n = 5), melon
Grains	17	26	Wheat (n = 32), buckwheat (n = 5), corn, oat, rye, barley
Milk	7	31	Cow milk (n = 36), goat milk (n = 2)
Egg	1	23	
Fish/shellfish	14	9	Tuna (high histamine; n = 5), perch, shrimp (n = 8), roe
Soy	6	3	Alpro soy drink (n = 4)
Other foods	30	14	Baked roll (n = 9), wine (n = 3), carmine red (n = 2), sweet (n = 2), yeast
Several foods	111	1	Milk-egg-wheat, a whole meal
Drugs	120	16	
Antibiotics	35	5	
Cephalosporin	12	2	Cefuroxime (n = 10), cefalexin (n = 2)
Penicillin	11	1	Phenoxymethyl penicillin (n = 9), filling agent macrogol (polyethylene glycol)
Other antibiotics	12	2	Amoxicillin (n = 5), macrolide (n = 4), fluoroquinolone (n = 2), trimethoprim (n = 2), tetracycline (n = 2), nitrofurantoin, chloramphenicol, metronidazole
Painkillers	28	10	
ASA	8	1	
Ibuprofen	5	3	Alone (n = 5) or with other drugs (n = 2)
Paracetamol	2	1	1 in a combination product
Other pain killers	11	2	Diclofenac (n = 4), naproxen (n = 6), coxib (n = 3), mefenamic acid
General anesthetics	25	0	
Rocuronium	17		Alone (n = 14) or with other anesthetics (n = 3)
Patent blue	3		
Other anesthetics	5		Several (antibiotic, propofol, fentanyl), gelatin
Local anesthetics	10	0	Lidocaine (vasovagal reaction), methylprednisolone of the product
Radiocontrast media	9	0	Iodobromide (n = 4), ioversol, ioxaglate, iomeprol (n = 2), iodixanol
Other drugs	22	2	Infliximab (n = 2), pollen product, insulin protamine, povidone, borophenylalanine, aurothiomalate, methylprednisolone (n = 3), angiotensin-converting enzyme inhibitor, reteplase, tetanus-diphtheria vaccine, antispasmodic, chlorhexidine
Allergen preparations	46	18	
SIT	37	17	
Timothy	32	13	Alone (n = 42) or with birch or grass mix
Other allergen	5	4	Birch (n = 6), cat (n = 2), dog, wasp
SPT	9	1	Egg, fish, penicillin, mould, carmine, chironomid, several allergens
Insects	25	15	
Wasp	19	14	
Other insects	6	1	Bee (n = 2), mosquito, chironomid, unknown (n = 2)
Other causes	11	2	Chemical, latex, viper bite, fragrance, algae, rabbit, plant, other (n = 5)
Not known	4	2	
All	348	182	In some cases, several causes were present

Hospitals and other health care settings were the most common venues for severe allergic reactions overall (33%), followed by the home (30%), hobbies (14%), restaurants (7%), day care/schools (7%), and work places (6%).

### Food

Food (n = 279) was the most common cause of severe allergic reaction (53%). Of these patients, 53% were adults, 64% of whom were females, but most (60%) of the children were boys. The home was the most common place for food reaction (36%).

Food was responsible for 72% of the reactions in children, but for only 43% in adults. An association with exercise was documented in 8% with food-related reactions. Food caused 46% of the most severe shock reactions, including peanut/tree nuts/seeds (n = 17), grains (n = 11), milk (n = 10), fruit/vegetable (n = 8), egg (n = 6), fish/shellfish (n = 2), soy, and yeast among others.

An allergen-containing food was accidentally given to 53 patients, 60% of whom were children. These included milk, wheat, egg, fish, nuts, and seeds. Most episodes (77%) took place outside the home.

Most food-related reactions were from peanuts, tree nuts, and seeds (21%), 36% of which occurred in children (Table 3). One adult experienced anaphylaxis after eating home-delivered Thai food that contained soft-treated peanuts, which the patient assumed were beans. Tree nuts were involved in 53% of the nut/seed cases. Hidden or nonrecognized nuts were the causative agent in bread and in ice cream. Two other patients (1 child and 1 adult) had anaphylaxis after eating tahini sauce not recognized by the patients as being a sesame seed product.

Fruits and vegetables were reported in 16% of the food anaphylaxis cases, of which kiwi, apple, and celery were the most common allergens. In 2 birch pollen-allergic adults, even the first ingestion of an exotic fruit such as jackfruit or lychee when abroad resulted in severe anaphylactic reactions.

Regarding grains, reports numbered 14% of the cases, 72% of which were from wheat. One wheat-allergic child had a severe reaction after eating gluten-free pasta, and another child after eating a wheat-free bun made of buckwheat to which the child had developed an allergy during a 3-year wheat-free diet. Buckwheat in a blin was unexpectedly allergenic to 1 adult patient, whereas another adult experienced anaphylaxis from a bun in which the marzipan was dyed with a red color from cochineal insects (carmine, E120).

**Figure 2:**
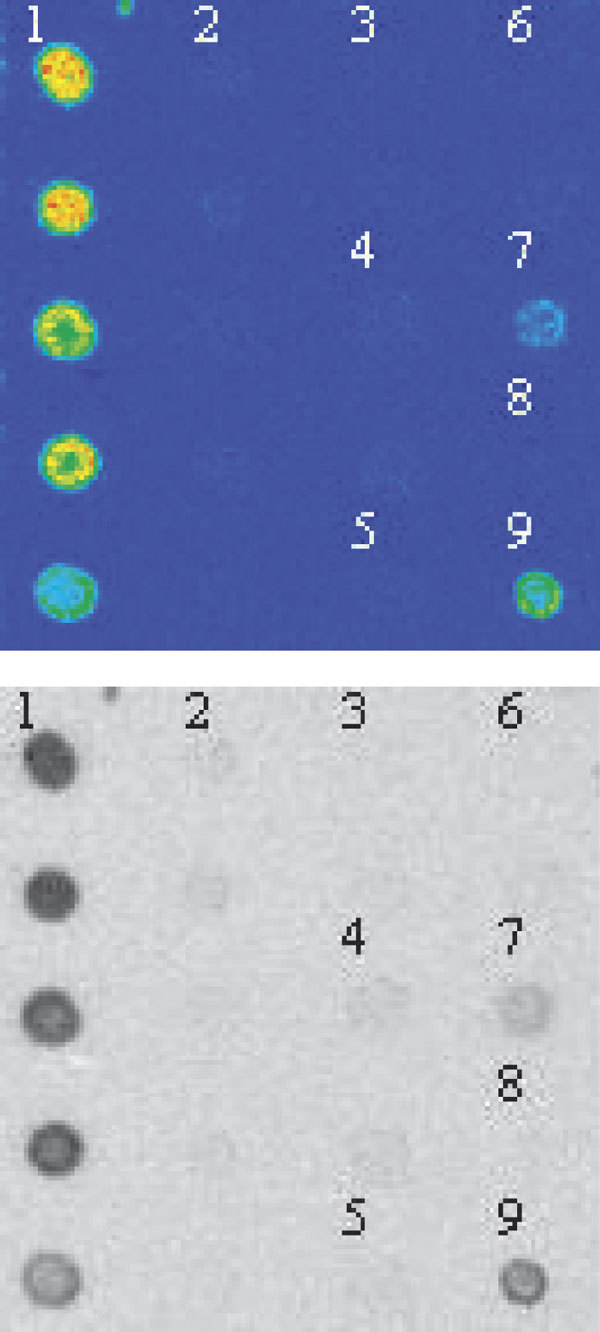
**In-house immunospot method demonstrates serum IgE to the inner part of the goat cheese delivered by the patient with anaphylaxis**,[**1**]**but not to cow's milk **[**8**]. Other examples are the mouldy surface of the goat cheese,[2] cow milk casein,[3] sheep milk casein,[4] carrageen used in the cheese,[5] cacao also suspected as an allergen,[6] egg white,[7] and goat cheese from the laboratory repertory [9].

Milk was the causative agent in 14% of the food-related anaphylaxis cases. Heavy exercise together with milk protein-containing pills caused a severe reaction in a milk-allergic adult to whom the terms *casein *and *whey *were unknown. The patient had an adrenaline autoinjector, but she did not dare use it. One adult experienced anaphylaxis from cow's milk feta cheese, but she tolerated goat's milk feta, whereas another adult experienced anaphylaxis from goat cheese without a cow's milk allergy (Figure 2).

Egg was reported in 9% of the food reactions, all but one in children, and 7 of which were accidents outside the home. In 1 case, a hidden allergen was suspected.

Fish or seafood was reported in 8% of the cases. Fish nuggets were given to a child instead of chicken nuggets (the Finnish word for fish is *kala*, for chicken, *kana*). Five reports were toxic reactions from tuna fish in which a high histamine content was suspected as the causative agent because no fish allergy was evident. Eight cases were from shrimp and one from roe in the absence of fish allergy [19].

Soy was reported or suspected in 9 cases, 4 of which occurred in birch pollen-allergic patients after a soy drink. A skin prick test with native soy bean was positive, whereas a commercial serum soy-specific IgE test was negative, indicating the missing labile cross-reactive soy allergen (Gly m 4) [20]. In other cases, soy was suspected as a hidden allergen in the food offered in day care centers.

Yeast used as a flavoring agent in a spaghetti sauce repeatedly caused severe anaphylactic reactions in a mould-sensitive patient [21].

Drinks were reported in 11 cases (4%), mostly (73%) in adults. Reported drinks included red and white wine, beer, long drink, cider, juice, and glühwein. One patient repeatedly experienced anaphylaxis when dancing the tango after drinking a beer.

### Drugs, Allergen Preparations, and Other Interventions

Drugs (n = 136), allergen preparations (n = 64), and other medical interventions such as food provocation tests (n = 22) were reported in 222 cases (42%), mostly (75%) in adults (Table 3).

Antibiotics in 29% and painkillers in 24% were the most commonly reported in drug-related reactions. Cephalosporins and penicillins comprised 58% of the antibiotics, whereas acetylsalicylic acid (ASA) and ibuprofen accounted for 45% of painkillers. In 4 cases, ASA in a combination product was not recognized by the patient as such.

In 68% of the operation-related reactions, a neuromuscular blocking agent (rocuronium) was suspected as the causative agent either alone or with other anesthetic agents. Three cases were caused by the patent blue dye (E 131) used to locate sentinel lymph nodes. One reaction was caused by chlorhexidine used as skin disinfectant [22]. Local anesthetics were reported in 8 cases, in one of which methylprednisolone used simultaneously proved to be the anaphylactic agent based on positive intradermal testing. Nine cases were from contrast media and one from iodinated povidone (Betadine).

In a drug, the active ingredient is not always the agent causing harm. One case of penicillin anaphylaxis was caused by macrogol (polyethylene glycol) used as an excipient in the tablet vehicle [23].

Allergen preparations were the causative agent in 64 cases (12%), 18 of which occurred in children. Ten reports with mild reactions were from skin prick testing with fish, egg, penicillin, moulds, pollens, animals, chironomid, or carmine dye (E120). Carmine IgE was positive in in-house immunospot test but remained negative in the commercial IgE test, which, however, was improved in collaboration with our laboratory.

Of all reports, 54 (10%) concerned specific immunotherapy (SIT). In 83% of the reactions, timothy grass was the causative allergen, either alone or with other pollen allergens. Nine patients were on a maintenance dose; some for several years.

Of the severe anaphylactic shock reactions, 45% were caused by drugs, allergen preparations, and other interventions, such as operations (n = 16), painkillers (n = 13; ASA, coxib, naproxen, ibuprofen), *β*-lactams (n = 11), timothy SIT (n = 5), x-ray contrast media (n = 5), infliximab, sodium aurothiomalate, and a natural remedy (pollen) among others.

### Insects and Other Agents

Insects were reported in 8% of the cases, 63% of which were in adults. Wasps (92%) were by far the most commonly reported. Insects caused 9% of the severe shock reactions.

## Discussion

Based on our study, during the 8 years, 530 severe allergic reactions were reported to the Finnish register for an annual incidence of 0.001%. Reactions are undoubtedly underreported, with higher figures per population reported elsewhere [10].

From the reactions, 26% were life-threatening circulatory or respiratory shock reactions. Adrenaline was the first aid treatment for 75%, but corticosteroids were more frequent: 84% of all cases. In a Swiss study, 48% of the anaphylaxis patients with circulatory symptoms received adrenaline,[9] whereas in an Italian study, only 6% of the patients did [24]. Finnish health care is thus reasonably well prepared to use adrenaline appropriately, but still with room for improvement.

In 5% of our cases, a late-phase reaction occurred compared with late reactions reported in 2% to 20% of cases elsewhere [13,25].

Food, drugs, and insects alternate globally as the most common causes of anaphylaxis [9,11,13,18,26]. In an overview from 1 referral clinic, 59% of the anaphylactic cases were considered idiopathic [27]. In our register, food was the most commonly reported cause (53%), and only 1% of the cases were without a known or highly suspect cause. Fruits, vegetables, nuts, and seeds were the most common causes for food reactions in adults. Similarly, fresh fruits and vegetables have been the most important allergens in adult anaphylaxis in Italy [24]. Thirteen (5%) of the food reactions involved peanut, a common cause for fatal anaphylaxis in the United States and United Kingdom, where the Anaphylaxis Campaign has also reported an increasing frequency/number of sesame anaphylaxis [14,28]. Two of our patients experienced anaphylaxis from sesame sauce tahini. Fish and shellfish were rather seldom reported (8%) but are common causes of anaphylaxis in Italy, Asia, and the United States [13,14,24,27]. No deaths from food are known in Finland.

Diagnostics and other interventions are not without risks [8-10,13,15,28,29]. Of our cases, 42% were from drugs, operations, diagnostics, and other interventions. In line with the reports of neuromuscular blocking agents as the most frequently implicated anesthetic drug, rocuronium was the causative agent in 68% of the surgical anaphylaxis cases [10,29,30].

Severe allergic reactions from allergy diagnostics (skin test and food/drug challenge test) and allergy treatment (SIT) comprised 18% of the cases, and a high frequency of SIT anaphylaxis has been reported only by Mehl et al. [26] Timothy pollen SIT, but not birch pollen SIT, seems to be associated with a relatively high risk for severe reactions, a fact practitioners giving SIT in Finland have been warned about.

Insects are important anaphylactic allergens in Switzerland and in the United Kingdom but account for only 8% of the reports in Finland, wasps being the most frequently reported [8,9]. Because commercial skin prick and serum IgE tests are not always sufficiently sensitive and specific to identify the sensitizing insect, for SIT assessment in our hospital, an in-house immunospot method with several venom samples differentiates between wasps and honey bees [21]. No deaths were reported, although such fatalities are estimated to occur approximately every second year in Finland.

We found only 1 report on severe reaction from latex, an important allergen in France and the United States [10,18,25,27,30].

To avoid is to identify. Our register has been proven to be valuable for identifying causes for severe allergic reactions in Finland. Thus far, poorly recognized allergens and allergen sources have been detected, such as patent blue, carmine, yeast, buckwheat, soy drink, and macrogol. In addition, hidden allergens have been demonstrated in food and drugs. In France, hidden allergens have accounted for 13% of severe anaphylactic reactions [10].

Our register has also contributed to first aid treatment practice by highlighting the importance of intramuscular adrenaline as first-line treatment.

All patients experiencing a severe allergic reaction should be sent to an allergologist who will evaluate the causative agent, provide guidance for avoidance and prevention, and also consider SIT. The patient, family members, and caregivers must be instructed on how to use an adrenaline autoinjector.
